# The Role of the *Moraxella catarrhalis* CopB Protein in Facilitating Iron Acquisition From Human Transferrin and Lactoferrin

**DOI:** 10.3389/fmicb.2021.714815

**Published:** 2021-09-23

**Authors:** Clement Chan, Dixon Ng, Anthony B. Schryvers

**Affiliations:** Department of Microbiology, Immunology and Infectious Diseases, Cumming School of Medicine, University of Calgary, Calgary, AB, Canada

**Keywords:** TonB dependent transporters, transferrin, lactoferrin, mechanism of transport, iron transport

## Abstract

*Moraxella catarrhalis* is a Gram-negative bacterium that is responsible for a substantial proportion of upper respiratory infections in children and lower respiratory infections in the elderly. *Moraxella catarrhalis* resides exclusively on the mucosal surfaces of the upper respiratory tract of humans and is capable of directly acquiring iron for growth from the host glycoproteins human transferrin (hTf) and human lactoferrin (hLf). The iron-bound form of these glycoproteins is initially captured by the surface lipoproteins Tf or Lf binding protein B (TbpB or LbpB) and delivered to the integral outer membrane TonB-dependent transport (TBDT) proteins, Tf binding protein A (TbpA) or Lf binding protein A (LbpA). The extraction of iron involves conformational changes in Lf and Tf to facilitate iron removal followed by its transport across the outer membrane by a well characterized process for TBDTs. Surprisingly the disruption of the gene encoding another TBDT, CopB, results in a reduction in the ability to grow on human Tf or Lf. The possibility that this could have been due to an artifact of mutant construction that resulted in the inhibition of TonB-mediated process was eliminated by a complete deletion of the CopB gene. A systematic evaluation of the impact on growth under various conditions by deletions of the genes encoding TbpA, LbpA, and CopB as well as mutations of the iron liganding residues and TonB box region of CopB was implemented. The results indicate that although CopB is capable of effectively acquiring iron from the growth medium, it does not directly acquire iron from Tf or Lf. We propose that the indirect effect on iron transport from Tf and Lf by CopB could possibly be explained by the association of TBDTs at gaps in the peptidoglycan layer that may enhance the efficiency of the process. This concept is supported by previous studies demonstrating an indirect effect on growth of Tf and Lf by deletion of the peptidoglycan binding outer membrane lipoprotein RmpM in *Neisseria* that also reduced the formation of larger complexes of TBDTs.

## Introduction

*Moraxella catarrhalis* is a Gram-negative bacterium that is an important cause of upper respiratory tract infections in young children and lower respiratory tract infections in elderly patients with chronic obstructive pulmonary disease (De Vries et al., 2009; Murphy and Parameswaran, 2009). However, it is also a common inhabitant of the upper respiratory tract, where it exclusively resides aside from being an occasional inhabitant of the human genitourinary tract. It is capable of directly utilizing transferrin (Tf) and lactoferrin (Lf) that are available on the mucosal surface by virtue of its surface receptors that can bind the host iron-binding proteins and extract the iron required for growth (Yu and Schryvers, 1993; Campagnari et al., 1994; Bonnah et al., 1998). This enables *M. catarrhalis* to directly access these proteins as a source of iron for growth and proliferate independently from other members of the microbial community that inhabit the upper respiratory tract. The critical role that these receptors play in the survival of *M. catarrhalis* led to preliminary studies evaluating their potential as vaccine antigens (Bonnah et al., 1998; Myers et al., 1998; Yu et al., 1999).

**Figure 1:**
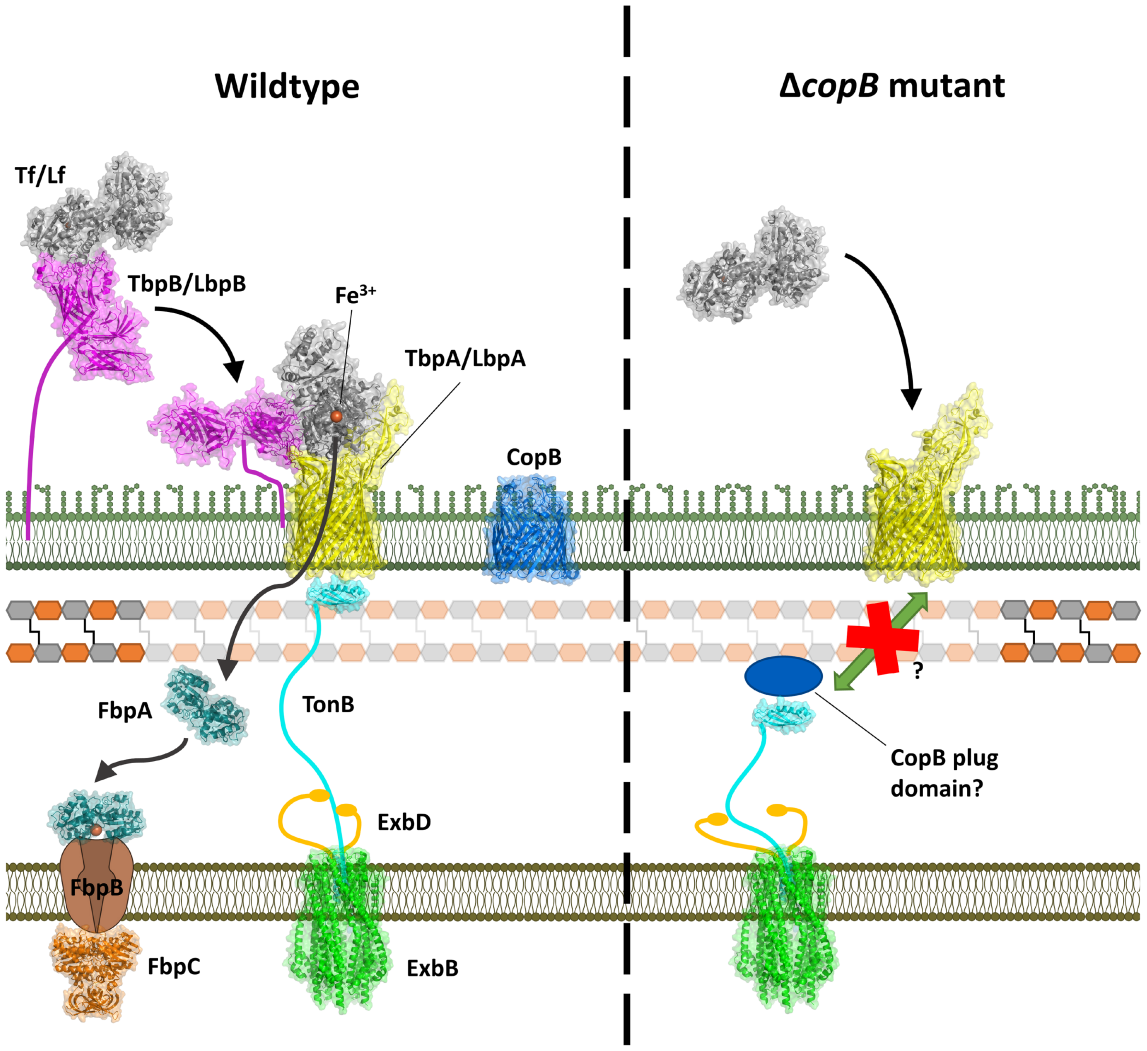
Model for iron acquisition from Tf or Lf and its inhibition by a CopB fragment. **Left panel**: after capture of iron-loaded Tf/Lf by TbpB/LbpB, it is transferred to TbpA/LbpA with concomitant domain separation of the C-lobe domains. Interaction with TonB provides energy for transport of ferric ion through the TbpA/LbpA barrel which is captured by FbpA and shuttled to the inner membrane transport complex. **Right panel**: an N-terminal fragment generated by the insertional activation of the *copB* gene would be expected to produce the entire CopB plug region and interfere with TonB-mediated transport.

The Tf and Lf receptors are comprised of two proteins, a surface accessible lipoprotein and an integral outer membrane protein (Morgenthau et al., 2013). The surface lipoproteins, Tf and Lf binding protein B (TbpB and LbpB) have an N-terminal anchoring peptide region that allows TbpB or LbpB to extend away from the surface of the outer membrane to capture the iron-loaded form of Tf or Lf (Moraes et al., 2009; Yang et al., 2011; Figure 1, left Panel). This is thought to be particularly important under conditions where the availability of the iron-loaded host proteins would be limited, such as on the mucosal surface. However, the lipoproteins are not essential for growth under laboratory conditions. The extraction of iron from Tf or Lf and the transport of iron across the outer membrane is mediated by the TonB-dependent transporter (TBDT) component of the receptor, Tf binding protein A (TbpA) or Lf binding protein A (LbpA), thus this protein is essential for iron acquisition under all conditions. Although the process by which TbpB brings iron-loaded Tf to TbpA is not fully understood, it likely involves conformational changes in the anchoring peptide region and an interaction between a portion of the anchor peptide and TbpA (Yang et al., 2011) so that a ternary TbpB-Tf-TbpA complex is formed for iron removal.

TbpA has structural features shared by all TBDTs, a 22-strand β barrel and an N-terminal plug domain that is inserted in the β-barrel that facilitates transport across the outer membrane. The binding of substrate by TBDTs results in conformational changes that result in unfolding of the N-terminal TonB box region making it accessible to TonB in the periplasmic space, and upon binding provides energy for conformation changes in the plug region that facilitate transport. This process is more complex for TbpA where binding of Tf involves substantial conformational changes that result in separation of the domains in the C-lobe to facilitate iron release, and a loop from the plug region is inserted into the gap that simultaneously causes unfolding of the TonB box region (Yadav et al., 2019). This process is thought to provide a path for iron to move through the beta-barrel, and hydrogen-deuterium exchange experiments suggest that the apo form of the periplasmic iron-binding protein, FbpA, may complex with TbpA for efficient transfer of the ferric ion (Siburt et al., 2009).

The mechanism of iron acquisition from Lf by LbpB and LbpA is thought to be equivalent to the process demonstrated with the TbpB-TbpA receptor, but has been more challenging to pursue due to the presence of a cationic region in the N-terminus of Lf and a surface loop enriched in aspartic and glutamic acid residues present in LbpB that protects the bacteria against the action of cationic antimicrobial peptides (Morgenthau et al., 2012, 2014). As a consequence, there are two distinct interactions between LbpB and Lf, one between the C-lobe of Lf and N-lobe of LbpB and another between the N-lobe of Lf and the C-lobe of LbpB (Ostan et al., 2017). This not only makes it challenging to probe these two interactions but has the tendency to generate larger protein complexes of LbpB and Lf.

Several years after the identification of the Tf and Lf receptor proteins from *M. catarrhalis* (Schryvers and Lee, 1989) CopB was identified as a major outer membrane protein and potential vaccine candidate from *M. catarrhalis* (Helminen et al., 1993a). However, subsequent studies demonstrated that the immune response in human convalescent sera was primarily associated with TbpB (Campagnari et al., 1996; Mathers et al., 1997), which may have reduced the interest in pursuing CopB as a vaccine candidate. In parallel, early studies identified what is now known as a CopB homologue, FrpB, as a major iron regulated outer membrane protein in *Neisseria meningitidis* (Ala'Aldeen et al., 1994). The demonstration that FrpB in *Neisseria gonorrheae* was capable of mediating iron acquisition from ferric-enterobactin and was part of an operon with a siderophore binding protein required for utilization (Carson et al., 1999) resulted in renaming it as FetA (ferric enterobactin transporter). The TBDT by FetA was subsequently shown to include an extended range of catecholate xenosiderophores (Hollander et al., 2011). *Neisseria gonorrheae* was also shown to use additional xenosiderophores in a TonB-independent pathway that relied on the periplasm to cytoplasm FbpABC uptake pathway (Strange et al., 2011). Another homologue of CopB present in strains of *Moraxella bovis*, IrpA, was shown to impact iron acquisition from bovine Tf and Lf, but not other ferric iron sources (Kakuda et al., 2003).

TonB-dependent substrate transport is normally described as a process where a single TBDT interacts with a single molecule of TonB to facilitate substrate uptake. This is consistent with the observation that deletion of the gene for a specific TBDT results in the loss of the ability to use a specific substrate as a source of metal ion for growth. Thus, the deletion of the gene encoding the Tf binding protein A from *M. catarrhalis* results in the inability to use human Tf (hTf) as a source of iron (Luke and Campagnari, 1999) and deletion of the gene encoding Lf binding protein A eliminates growth on human Lf (hLf) as an iron source (Bonnah et al., 1999). However, the disruption of the *copB* gene that encodes a TBDT that has not been shown to bind to either substrate reduces growth on these two distinct iron sources (Aebi et al., 1996).

TbpAs and LbpAs from bacterial pathogens are exquisitely specific for Tfs and Lfs from their mammalian host (Morgenthau et al., 2013) and clearly specifically bind only to Tf or Lf. The direct removal of iron from Tf and Lf by TbpA and LbpA not only requires direct binding of the host protein but separation of the two domains of the C-lobe mediated by conformation changes induced by the specific interactions with the two C-lobe domains (Noinaj et al., 2012). Since it is unlikely that CopB would be capable of directly removing iron from hTf and hLf, it raised the question whether the observed impact of CopB on iron acquisition from Tf and Lf (Aebi et al., 1996) could be an unexpected consequence of the insertional inactivation of the *copB* gene (Figure 1, right Panel). The resulting N-terminal plug region of CopB could be interacting with TonB through its “TonB box” and reducing TonB-mediated transport. In this study, we demonstrate that complete removal of the CopB gene does reduce growth with exogenous hTf or hLf, excluding the mechanism illustrated in Figure 1, and evaluate aspects of the TonB-mediated process that may contribute to this phenomenon.

## Materials and Methods

### Bacterial Strains and Growth Conditions

The bacterial strains that were used in this study are listed in Table 1. *Moraxella catarrhalis* strain O35E (N148) and the O35E Δ*copB* mutant has been previously described elsewhere in detail (Helminen et al., 1993a,b; Aebi et al., 1996). The single knockout strains in this study (N346, N482, and N486) were generated by preparing linear DNA constructs with splicing by overlap extension PCR (SOE-PCR) and incorporating the DNA into the bacterial genome through homologous recombination. The chloramphenicol or kanamycin resistance regions were incorporated in between the upstream and downstream regions for production of the gene knockouts.

**Table 1:** List of the bacterial strains in this study.

Strain	Genotype	Source
*M. catarrhalis*		
N148	O35E Wild type	Helminen et al., 1993b
N149	N148 *copB::kan* (Insertion of KanR within *copB* gene)	Helminen et al., 1993b
N346	N148 *copB::cat* (Complete removal of *copB*)	This study
N503	N148 Containing CopB WT, KanR	This study
N443	N148 Containing CopB Δ12-16, KanR	This study
N446	N148 Containing CopB V14P, KanR	This study
N448	N148 Containing CopB V15P, KanR	This study
N466	N148 Containing CopB with extracellular loop 3 transplantation of O35E CopB with 417:082 CopB loop 3, KanR	This study
N470	N148 Containing CopB H89A, KanR	This study
N478	N148 Containing CopB H89A Y355F, KanR	This study
N480	N148 Containing CopB Y355F, KanR	This study
N482	N148 *tbpA::kan*	This study
N484	N148 *tbpA::kan; copB::cat*	This study
N486	N148 *lbpA::kan*	This study
N488	N148 *lbpA::kan; copB::cat*	This study
N491	N148 REEF mutant, KanR	This study
N492	N148 Containing CopB with extracellular loop 3 replaced with short linker peptide, KanR	This study
N494	N484 Containing *tbpA* in *copB* loci, GmR	This study
N498	N488 Containing *lbpA* in *copB* loci, GmR	This study

The CopB-TonB box mutant strains (N443, N446, and N448), the CopB iron binding mutant strains (N470, N478, and N480) and the CopB REEF and loop 3 variant strains (N466, N491, and N492) were prepared by SOE PCR using the primers listed in Table 2 and assembling the modified *copB* gene with a downstream KanR region between the upstream and downstream regions. The introduction of KanR downstream of the *copB* locus has been shown to not affect hTf/hLf iron utilization phenotypes. The assembled DNA segment was used to transform strain N346, replacing the chloramphenicol resistance cassette with the modified *copB* gene and kanamycin resistance cassette.

**Table 2:** Table of the mutagenic oligonucleotide primers used in this study.

Mutation	Oligonucleotide primers
Δ12-16	5′CCTAAGGTTGTCTTGGCAGGC***GGT***GATCGCCAAGGTGCAAAAATT3′ 3′GGATTCCAACAGAACCGTCCG***CCA***CTAGCGGTTCCACGTTTTTAA5′
V14P	5′CCTAAGGTTGTCTTGGCAGGCGATACA***CCT***GTCAGTGATCGCCAAGGTGCAAAAATT3′ 3′GGATTCCAACAGAACCGTCCGCTATGT***GGA***CAGTCACTAGCGGTTCCACGTTTTTAA5′
V15P	5′CCTAAGGTTGTCTTGGCAGGCGATACAGTG***CCT***AGTGATCGCCAAGGTGCAAAAATT3′ 3′GGATTCCAACAGAACCGTCCGCTATGTCAC***GGA***TCACTAGCGGTTCCACGTTTTTAA5′
H89A	5′GGTCAATTACACTAC***GCA***CAAGGTCGCTTTATGCTAGACCCCCAG3′ 3′CTGTTGCGAATAGTTCTACCAGTTAATGTGATG***CGT***GTTCCAGCGAAATAC5′
Y355F	5′AGCGATAAAGGTAACAGC***TTT***ATTCTTCGTGATGTACCTAATACCATCAATGATAAC3′ 3′GCGTTGAGGAGATCGCTATTTCCATTGTCG***AAA***TAAGAAGCACTACATGGA5′
Loop 3 from 417:082	5′***GTTAACTTTGATCTTAAACGCCAAGCGCCACAT***CAGCGTGAAACCTACCAAAAGTTAACCAAC3′ 3′TTTCCGTAAGCACCGCACGCACTTCTCAAA***CAATTGAAACTAGAATTTGCGGTTCGCGGTGTA***5′
REEF domain mutation	5′GGCGTG***GGCGCAGCTGGT***GACTTCGCCAATCGTGCCTTGACG3′ 3′CTTCAAGTATTTCCGTAAGCACCGCAC***CCGCGTCGACCA***CTGAAGCGG5′
Loop 3 with synthetic peptide	5′GGC***GCAGGTTCATCCGCAGGTGGC***CCAACTCAGCGTGAAACCTACCAAAAGTTA3′ 3′TTACTTCAAGTATTTCCGTAAGCACCG***CGTCCAAGTAGGCGTCCACCG***GGT5′

Mutations that were introduced into the oligonucleotide primers are in bold and italicized.

The double mutant strains N484 and N488 were prepared using the genomic DNA/PCR product from N346 to transform N482 and N486, respectively. These strains in turn were used to generate strains N494 and N498 by assembling the *tbpA* or *lbpA* genes with a downstream gentamicin resistance cassette between the upstream and downstream regions from the *copB* gene thus replacing the chloramphenicol cassette.

*Moraxella catarrhalis* strains were either grown on chocolate agar or brain heart infusion (BHI) agar overnight at 37°C with 5% CO_2_, or on BHI broth at 37°C shaking at 230 RPM in a shaking incubator. To grow the bacteria in iron-deplete conditions or to induce the expression of iron-repressible outer membrane proteins, Desferal was added to the growth medium at a concentration of 50μM for broth or 50μg/ml for agar. For strains containing resistance genes, the antibiotics were added to the growth media at 20μg/ml for kanamycin, 1μg/ml for chloramphenicol, or 2μg/ml for gentamicin.

### Natural Transformation

The bacteria were grown to mid-log phase (OD600 of ~0.4–0.5) in BHI broth at 37°C using a shaking incubator. The cells were centrifuged at 5,939 x *g* for 1min at 4°C and the spent media was decanted. The cell pellet was then resuspended in 50μl of the PCR reaction mixture containing the gene knockout or site-directed mutation construct. The cell mixture was spotted onto a chocolate agar plate and was incubated overnight at 37°C with 5% CO_2_. The cells were resuspended in BHI the next day and serial dilutions were made. The diluted cells were plated on BHI agar containing 1μg/ml of chloramphenicol, 20μg/ml of kanamycin, or 2μg/ml of gentamicin. Positive colonies were screened for and verified by PCR and Sanger sequencing.

### Verification of CopB Protein Expression

The bacterial strains were first grown on chocolate agar overnight at 37°C with 5% CO_2_ in presence of either 20μg/ml kanamycin, 1μg/ml chloramphenicol, or 2μg/ml gentamicin. The bacteria were then used for inoculating BHI broth which was grown overnight in a shaking incubator at 37°C.

The culture was diluted the next day to an OD600 of 0.05 and 50μM of Desferal (Sigma) was added to the diluted culture, which was then incubated overnight in a shaking incubator at 37°C. The culture was centrifuged at 3,220 x *g* for 10min and the cell pellet was washed in PBS for three times. The cell pellet was resuspended in 2ml of 2% Elugent (Calbiochem)/PBS and was incubated at 4°C overnight on a tube rotator. The detergent-treated cell mixture was centrifuged at 16,000 x *g* for 10min to pellet any insoluble material. Twenty microliters of the supernatant was mixed 1:1 with LDS sample buffer and was loaded on to 4–12% SDS-PAGE gel. The gel was run at 180V for 30min and was visualized by Coomassie Blue staining.

### Purification of Tf/Lf Preparations

Tf and Lf were prepared by dissolving holo hTf (Sigma) or holo hLf (Sigma) in 50mM HEPES pH 8.0, 50mM NaCl. Size exclusion chromatography was performed by injecting the proteins into an ENrich SEC 650 column (Bio-Rad) using the NGC Quest 10 chromatography system (Bio-Rad) at 1ml/min. The eluted proteins were collected and concentrated to 50mg/ml using Vivaspin 20 with a 50kDa cut-off (GE healthcare).

### Iron Starvation of Bacteria

The bacterial strains were grown on chocolate agar overnight at 37°C with 5% CO_2_ in the presence of either 20μg/ml kanamycin, 1μg/ml chloramphenicol, or 2μg/ml gentamicin. Bacterial strains were grown overnight in BHI broth or BHI broth containing 50μM Desferal for comparison between growth in iron replete or deplete conditions. The cultures were diluted to OD600 of 0.1 the following day in BHI broth or BHI broth containing 50μM Desferal, respectively, and were grown at 37°C for ~6.5h in a shaking incubator. The cultures were then diluted to OD600 of 0.05 and 100μl from each of the diluted cultures was spread onto the BHI agar plates for the plate-based feeding assay.

### Plate-Based Feeding Assay

The bacterial strains were first grown on chocolate agar overnight at 37°C with 5% CO_2_ in presence of either 20μg/ml kanamycin, 1μg/ml chloramphenicol, or 2μg/ml gentamicin. Bacterial strains were grown in BHI broth to log phase at 37°C in a shaking incubator unless iron starvation was required for the experiment. The liquid culture was then diluted with BHI to an OD600 of 0.05, and 100μl of the diluted culture was spread onto BHI plates containing 10, 20, 30, 40, or 50μg/ml Desferal. Unless further purification was required, Tf and Lf were prepared by dissolving holo hTf (Sigma), holo hLf (Sigma) or holo pTf (Gibco) in 50mM HEPES pH 8.0, 50mM NaCl and the protein concentration was then adjusted to 50mg/ml. Five microliters of each of the iron sources was spotted onto the BHI agar with Desferal plate and the after the liquid was no longer visible the plate was incubated at 37°C with 5% CO_2_. Strains were compared to the wild type and the CopB knockout strain to determine the relative amount of growth on hTf/hLf.

### Growth Quantification of Plate-Based Feeding Assay

To further characterize the three growth levels from the plate-based feeding assay, bacterial growth from each growth area was resuspended in 1ml of PBS using a 10μl inoculation loop and the OD600 of the resuspended cells was measured using Ultrospec 10 cell density meter (Amersham Biosciences). Statistical analysis was performed using GraphPad Prism 8 software. Results are presented as mean±SEM. Data were analysed using two-tailed Student’s *t*-test.

### Homology Modelling of CopB

The CopB protein sequence from *M. catarrhalis* strain O35E was obtained from NCBI. The CopB signal peptide was predicted using SignalP 5.0 and was removed from the protein sequence (Almagro Armenteros et al., 2019). The crystal structure of the iron-loaded *N. meningitidis* FetA (PDB: 4AIQ) was used as template for homology modelling with SWISS-Model (Waterhouse et al., 2018). The quality of the output protein model was assessed using MolProbity to check for Ramachandran outliers and protein geometry (Williams et al., 2018).

## Results

### Implementation of the Plate Growth Assay

Our first step was to implement a growth assay that would be robust, to some extent emulate the conditions on the mucosal surface, and ideally be somewhat independent from the effect of the level of iron stores in the different strains being used. The conventional plate bioassays that involve applying the iron source onto sterile disks or into the wells punched into the agar (Hollander et al., 2011) result in a limited area for observing the growth outside of the disk or well. Thus, we implemented a plate bioassay in which the iron-containing solution was simply applied to the surface of the plate containing an iron chelator (Desferal) so that the visible zone of growth was larger.

Growth assays were performed under a variety of conditions with different mutant strains providing reproducible results when categorizing the bacterial growth as ++ (maximum or wild type growth), + (intermediate or < wild type growth) or − (no growth). This assay enabled us to explore a range of growth conditions with the various mutant strains to gain a more comprehensive appreciation of the mutations on the growth phenotype. In order to provide a more quantitative means of comparing the growth differences in selected experiments, we decided to resuspend cells from the area of growth and measure the OD600, a relatively simple procedure that provides an indirect estimate of cell count. To evaluate the reproducibility and reliability of the method, we performed five to six replicates of selected experiments using BHI plates with 50μg/ml Desferal and determined the statistical significance for the comparisons being made.

**Figure 2:**
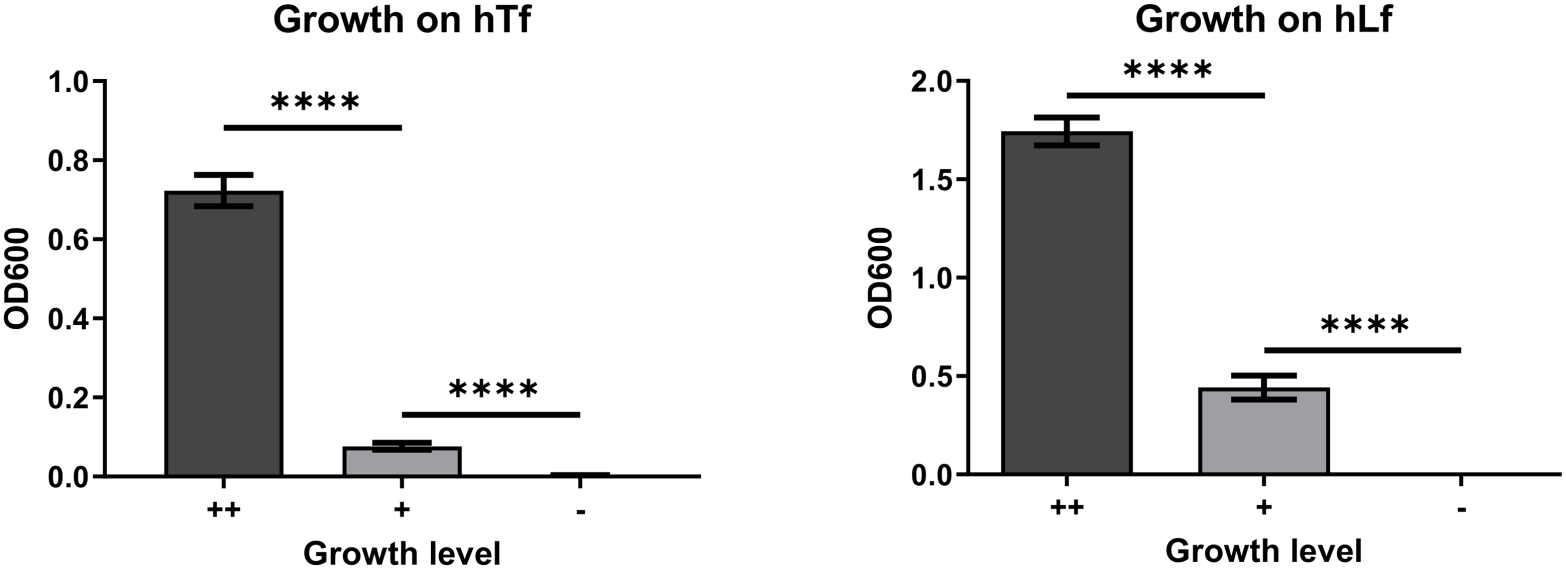
OD600 of resuspended cells from the bacterial growth on human transferrin (hTf) and human lactoferrin (hLf) in the plate-based feeding assay. ++, denotes wild type growth level; +, denotes less than wild type growth level; −, denotes no growth. The hTf experiment was performed using strains N148, N494, and N149; the hLf experiment was performed using strains N148, N149, and N488. Results are presented as mean±SEM. Each bar represents six replicates. ^****^Denotes *p*<0.0001.

The first experiment compared the growth of wild type and mutant strains with exogenous hTf or hLf and we selected mutant strains that would provide intermediate or no growth based on previous results (Figure 2). The ++ growth on hTf by the wild type strain (N148) was statistically greater than the + growth of a strain that had a mutation in CopB (N494) which in turn was statistically greater than the lack of growth observed for the insertional CopB defective mutant (N149). Similarly, the ++ growth on hLf by the wild type strain was statistically greater than the + growth by the insertional mutant strain (N149) which was statistically greater than the – growth by a strain lacking LbpA and CopB (N488). These results also illustrate that there is more growth observed with hLf as an exogenous iron source on media containing 50μg/ml Desferal, which is likely a consequence of its recognized ability to more effectively complex iron at low pH or sites of inflammation (Baker and Baker, 2004).

**Figure 3:**
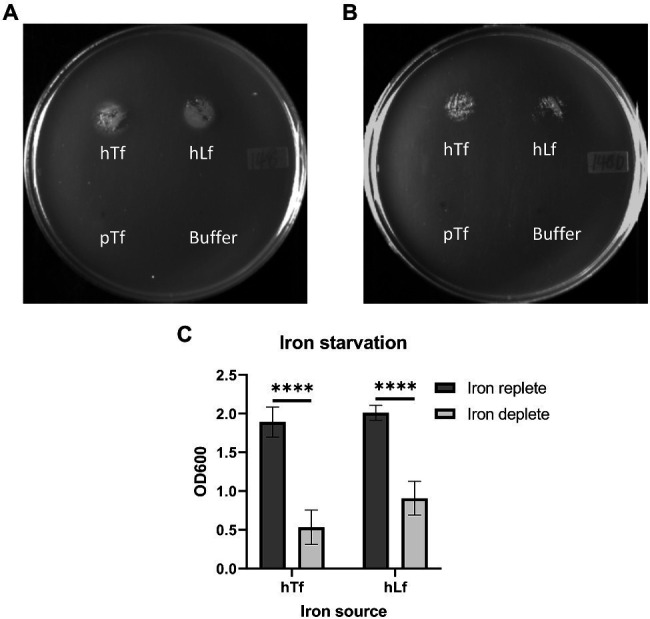
Effect of iron starvation on the growth phenotype of the wild type strain on Tf and Lf as the sole iron source. *Moraxella catarrhalis* wild type (N148) that was either grown in iron-replete (Panel **A**) or iron-depleted (Panel **B**) conditions was plated onto BHI agar with 50 ug/ml of Desferal. A 5μl solution alone or containing 3.1 nmole of hTf, hLf or porcine Tf (pTf) was spotted onto the plate and incubated overnight for growth. OD600 quantification of bacterial growth from the plate assay (Panel **C**). Results are presented as mean±SEM. Each bar represents five replicates. ^****^Denotes *p*<0.0001.

The growth dependent upon exogenous iron sources has a unique characteristic in that until the intracellular iron stores are largely depleted, there is limited ability to demonstrate differences in the ability of strains to acquire iron from exogenous iron sources. A standard inoculation of broth cultures of BHI and BHI with 50μM Desferal will not show any difference in growth until a second round is initiated by subculture. In order to determine the degree to which depletion of iron stores would impact the plate assay, wild type cells grown in iron replete (BHI) or iron-deplete (BHI with 50μM Desferal) media were used in the plate assay (Figure 3). The results demonstrate that the plate assay is quite robust in that both iron-sufficient and iron-depleted cells are able to achieve visible growth on the plate growth assay but that iron-deficient cells achieve a significantly lower level of growth than iron-sufficient cells.

### Does the Complete Loss of CopB Impact the Growth on hTf and hLf?

The insertional inactivation of the *copB* gene in a previous study that impaired the ability of *M. catarrhalis* to utilize hTf and hLf as the sole iron source (Aebi et al., 1996) would have resulted in the production and secretion of the entire N-terminal plug region of CopB into the periplasmic space. This raised the prospect of the plug region complexing with TonB, thus interfering with TBDT and explaining the impact on transport from Tf and Lf (Figure 1, right panel). To determine whether this was the explanation for the effect of CopB, we compared the original O35E parent strain and mutant from the published study with an additional mutant strain in which the coding sequence of the *copB* gene was removed and replaced by a chloramphenicol resistance cassette (Table 1). To test the impact of CopB on the growth of the wild type (N148), insertional CopB mutant (N149) and mutant with complete deletion of the *copB* gene (N346) on exogenous iron sources, preparations of hTf, hLf, and porcine Tf (pTf) were spotted onto the agar surface and assessed after overnight growth.

**Figure 4:**
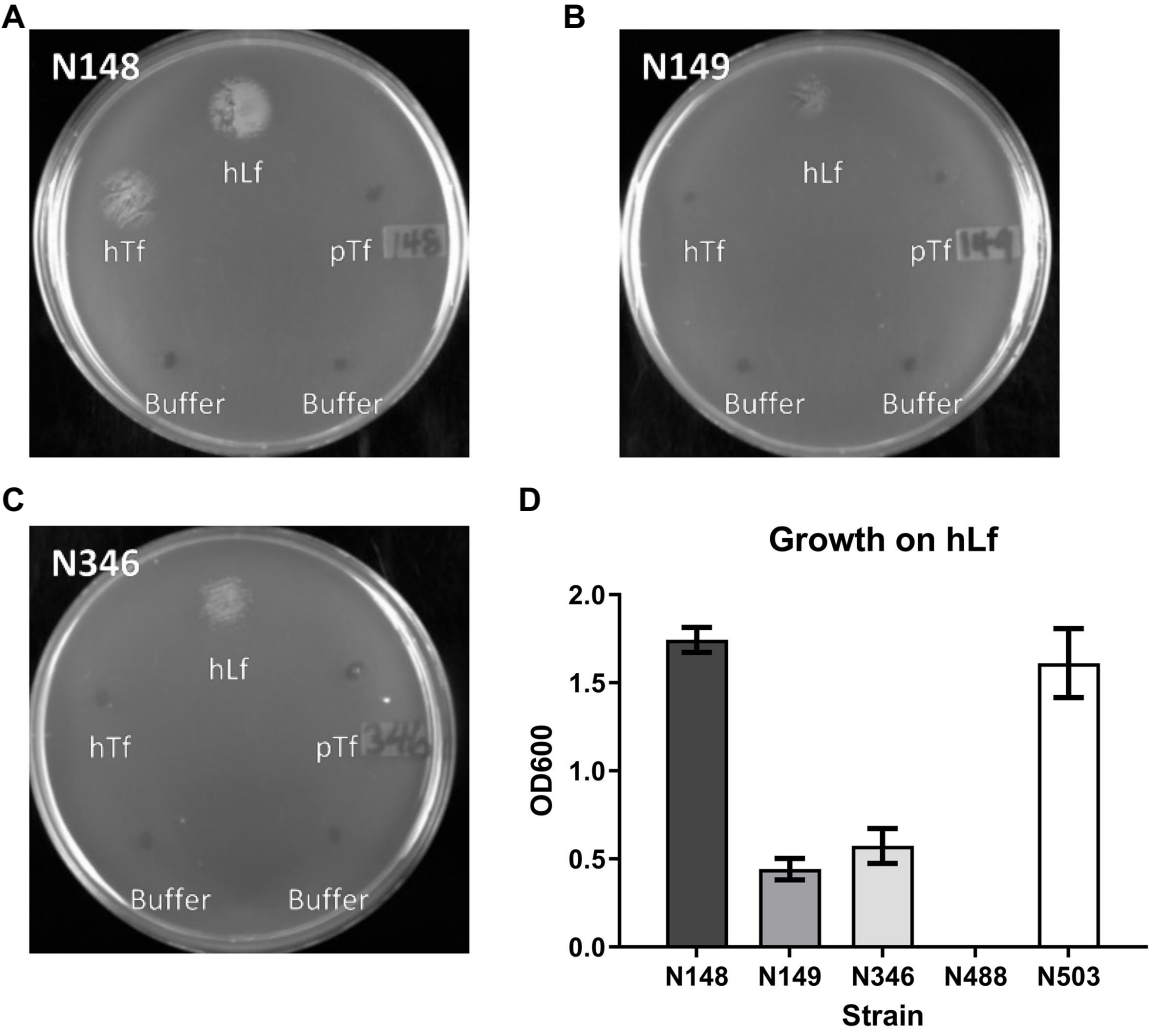
Growth of wild type and CopB mutant strains on transferrin and lactoferrin as sole iron source. *Moraxella catarrhalis* strain wild type (N148) (Panel **A**), insertional *copB* mutant (N149) (Panel **B**), and Δ*copB* mutant (*copB* gene completely removed – N346) (Panel **C**) were plated onto BHI agar with 50μg/ml Desferal. Five microliters of buffer alone or containing 3.1nmol of hTf, hLf or pTf was spotted onto the plate prior to incubating overnight for growth. OD600 quantification of bacterial growth on hLf from the plate assay (Panel **D**) in strains N148, N149, N346, Δ*copB*Δ*lbpA* mutant (N488), and Δ*copB* mutant complemented with wild type *copB* gene (N503). Results are presented as mean±SEM. Each bar represents six replicates.

The wild type strain (N148) grew on hTf whereas there was no growth observed for the two different CopB mutants (N149 and N346; Figure 4) indicating that the mechanism illustrated in Figure 1 is not solely responsible for the observed lack of growth. In contrast, there was some visible growth by the two mutant strains with exogenous hLf, but at a reduced level compared to the wild type. Since hLf is known to retain binding of the ferric ion under lowering pH conditions (Baker and Baker, 2004) and presumed to be more effective at sites of inflammation where it is released from neutrophils, it may be that the ability of hLf to retain binding of iron longer in the presence of Desferal is responsible for the reduced impact of CopB on uptake from hLf. Although visual inspection of the plates seemed to suggest that there might be more growth with the complete deletion of the CopB gene (N346) that with the insertional mutation (N149), there was not a significant difference when the quantitative assay was performed. Thus, the experimental data do not support the mechanism illustrated in Table 1. The reintroduction of wild type *copB* gene along with the kanamycin resistance cassette back into the *copB locus* of the Δ*copB* mutant (N503) restored a wild type-like growth phenotype (Figure 4D and data not shown) suggesting that the presence of a selection marker does not significantly affect the bacteria’s ability to utilize Tf and Lf as an iron source.

### Determining the Roles of CopB, TbpA, and LbpA on Uptake of Iron From Tf and Lf

The Δ*tbpA* (N482), Δ*lbpA* (N486), Δ*copB*Δ*tbpA* (N484) and Δ*copB*Δ*lbp*A (N488) mutants were made to evaluate their relative contribution to utilization of iron from Tf and Lf. The mutants were tested for their ability to grow on these protein sources of iron in BHI media with increasing concentrations of the iron chelator Desferal (Table 3). Lowering the Desferal concentration increases the risk of non-specific growth on trace iron contaminants. To minimize the trace iron contaminants in commercial Tf and Lf preparations, the Tf and Lf used in this experiment were further purified using size exclusion chromatography.

**Table 3:** Human transferrin, hLf, and pTf utilization phenotypes of wild type and knockout strains.

Iron source	Desferal (μg/ml)	WT	Δ*copB*	Δ*copB*Δ*tbpA*	Δ*copB*Δ*lbpA*	Δ*tbpA*	Δ*lbpA*
hTf	10	OG	++	++	++	OG	OG
	20	++	+	−	+	++	++
	30	++	+	−	+	−	++
	40	++	−	−	−	−	++
	50	++	−	−	−	−	++
hLf	10	OG	++	++	−	OG	OG
	20	++	+	+	−	++	−
	30	++	+	+	−	++	−
	40	++	+	+	−	++	−
	50	++	+	+	−	++	−

++, denotes wild type growth level; +, denotes less than wild type growth level; −, denotes no growth; OG, denotes overgrowth or a thick lawn of bacteria throughout the plate, not limited to the site of the exogenous iron source. The strains were either plated onto BHI agar with 50, 40, 30, 20, or 10μg/ml of Desferal. Five microliters of buffer alone or with 3.1nmol of hTf or hLf was spotted onto the plate.

When wild type cells or the cells lacking TbpA or LbpA were plated onto BHI plates with 10μg/ml of Desferal, they produced a thick lawn of growth (OG; Table 3) that clearly was independent of an exogenous iron source. In contrast, at this level of Desferal the strains lacking CopB did not produce a lawn of growth indicating that CopB is required for utilization of iron sources that are not fully complexed by Desferal at these concentrations. Under aerobic conditions it is expected that there will be clusters of ferric hydroxide ions that may not fully solubilized at these levels of Desferal particularly since the iron source is directly applied on top of the cells that are spread onto the plate. These results suggest that CopB is capable and required for utilizing these forms of iron for growth.

Although the Δ*copB* mutant strains did not form a thick lawn on the BHI plus 10μg/ml Desferal plates they were near the threshold for a visible lawn such that a small amount of iron would result visible growth. Thus, even trace amounts of iron released from the hTf preparation in the Δ*copB*Δ*tbpA* mutant was able achieve visible growth due to other iron acquisition pathways. In contrast, when hLf was provided as the iron source, growth only occurred in the mutant strains with LbpA present, indicating that iron could not be released from hLf. Since hLf is known to bind and retain iron under a broader range of conditions (i.e., low pH), it is not unexpected that growth with exogenous hLf would require direct removal by LbpA.

As expected, the Δ*lbpA* mutation had no impact on the growth on hTf as an iron source. It had an identical pattern of growth on varying levels of Desferal as the wild type strain, and the Δ*copB*Δ*lbpA* mutant had an identical pattern as the Δ*copB* mutant. The Δ*copB*Δ*tbpA* mutant was unable to grow on hTf at Desferal levels of 20μg/ml and above, the *tbpA* mutant was unable to grow at levels of 30μg/ml and the Δ*copB* mutant was unable to grow at 40μg/ml and above, confirming that CopB enhances the ability of TbpA to utilize iron complexed by hTf up to at least 50μg/ml.

For growth on hLf as an iron source the Δ*tbpA* mutation had no impact on the wild type or *copB* mutant strains. The *ΔcopB* mutation had a similar impact on the growth on hLf at increasing levels of Desferal up to at least 50μg/ml, whereas no growth was observed above 10μg/ml without LbpA being present. Since CopB was unable to acquire iron from hLf at levels above 10μg/ml without LbpA, it is unlikely that its ability to dramatically enhance the acquisition of iron from hLf at levels up to at least 50μg/ml could be due to directly acquiring the iron from hLf. The logical inference is that CopB somehow enhances the efficiency of iron acquisition by LbpA. Enhancement of iron acquisition by TbpA by CopB also provides a logical explanation for the results observed with hTf as an iron source.

**Figure 5:**
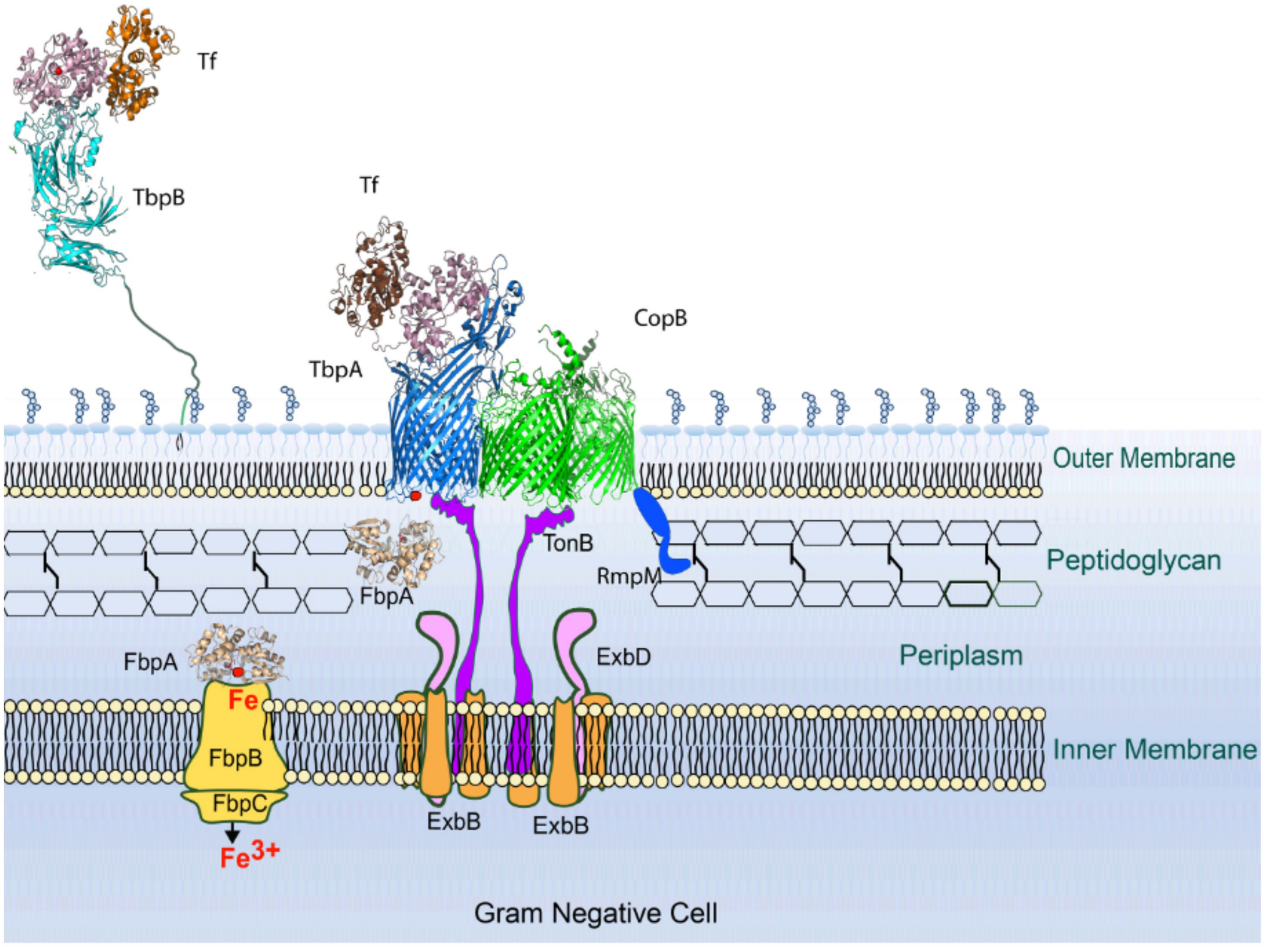
Model for TonB-mediated interactions in *M. catarrhalis*. In either newly formed or pre-existing gaps in the peptidoglycan layer, CopB is the dominant TonB-dependent transport (TBDT) present due to its high level of expression. Ultimately the TonB complex and TBDTs will cluster around the gap in the peptidoglycan layer resulting in more efficient transport. It is likely that an RmpM homologue will contribute to formation of the “clustered” TBDTs and indirectly influence TonB-dependent transport.

In an effort to provide possible mechanisms for the effect of CopB on the efficiency of iron acquisition from hTf and hLf, we drew on two important considerations: (i) CopB appears to be expressed at much higher levels than any other iron-regulated TBDTs in the outer membrane (Campagnari et al., 1994; Aebi et al., 1996), and (ii) the interaction of TonB with the TBDTs would be prevented by an intact peptidoglycan layer thus would only occur where there are gaps in the peptidoglycan layer (Figure 5). Since it is unlikely that gaps in the peptidoglycan layer are generated for each individual TonB-mediated transport interaction, a dominant (highly expressed) TBDT could play an important role in initiating or maintaining TonB interactions at gaps in the peptidoglycan layer, and perhaps directly or indirectly facilitate the association of TBDTs.

### Increased Expression of TbpA or LbpA Cannot Replace CopB Function

In an attempt to test whether it was merely the high expression level of CopB that was responsible for its role in enhancing the efficiency of iron acquisition from hTf and hLf, the *tbpA* or *lbpA* gene was expressed in the *copB* locus. An exogenous copy of the *tbpA* or *lbpA* gene was inserted into the *copB* loci of the Δ*copB*Δ*tbpA* and Δ*copB*Δ*lbpA* mutants. The resulting strains were evaluated using the feeding assay (Table 4). Insertion of the *lbpA* gene into the *copB* locus in the Δ*copB*Δ*lbpA* mutant strain did not restore growth on hTf to wild type levels demonstrating that LbpA cannot functionally replace CopB. Similarly, the *tbpA* gene inserted into the *copB* locus in the Δ*copB*Δ*tbpA* strain did not restore growth on hLf to wild type levels, indicating TbpA could not functionally replace CopB. A logical follow-up to these experiments would be to screen a variety of different TBDTs to replace CopB, including homologues from other species, as it might provide insights as to what attributes of CopB are responsible for this phenomenon.

**Table 4:** Growth phenotypes on exogenous hTf and hLf of strains generated by insertion of tbpA in copB locus of the ΔcopBΔtbpA mutant and insertion of lbpA in copB locus of the ΔcopBΔlbpA mutant.

Iron source	WT	Δ*copB*	*tbpA* in *copB* locus	*lbpA* in *copB* locus
hTf	++	−	+	−
hLf	++	+	+	+

++, denotes wild type growth level; +, denotes less than wild type growth level; −, denotes no growth. The strains were either plated onto BHI agar with 50μg/ml of Desferal. Around 3.1nmol of hTf, hLf, pTf, and buffer control was spotted onto the plate.

### The Role of Iron-Binding Residues in CopB Function

**Figure 6:**
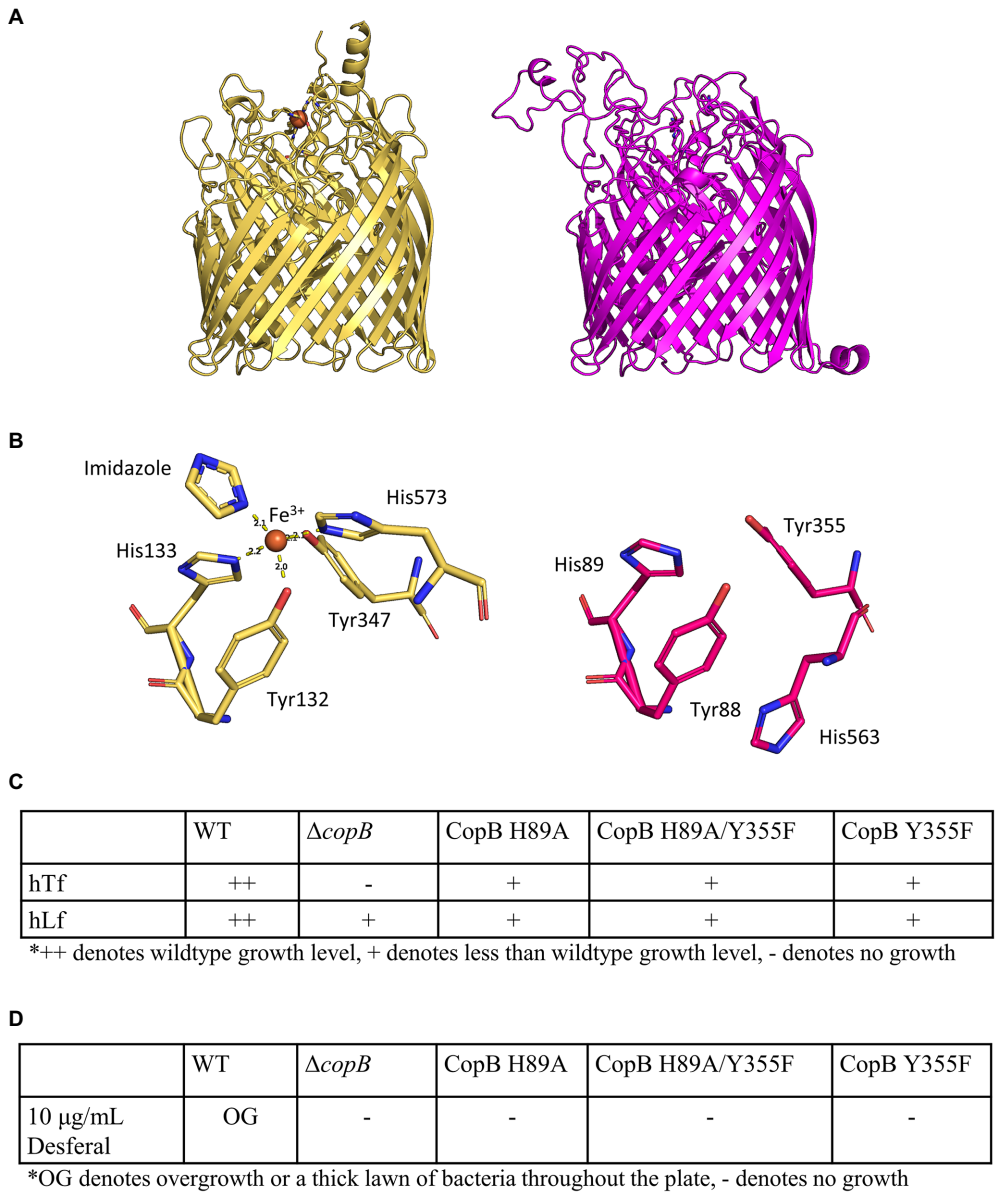
Impact of iron binding mutants of CopB on growth. **(A)** Crystal structure of iron-holo *Neisseria meningitidis* FetA (PDB ID: 4AIQ) (tan) and homology model of *M. catarrhalis* CopB (magenta). **(B)** *Neisseria meningitidis* FetA iron-binding residues (tan) and putative iron-binding residues (magenta). **(C)** Growth phenotypes of CopB iron-binding residue mutants on BHI plus 50μg/ml Desferal with hTf or hLf as sole iron source. **(D)** Growth phenotypes of CopB iron-binding residue mutants on BHI plus 10μg/ml Desferal without any exogenous iron sources.

The coordination of ferric ion in the CopB homologue, *N. meningitidis* FrpB (FetA), has been resolved in the crystal structure of the protein (Figures 6A,B; Saleem et al., 2013). As CopB has sequence identity of 45% with FrpB, we used the iron-holo FrpB structure (PDB ID: 4AIQ) as the template for the computational modelling of CopB. A comparison with the FrpB iron-binding residues showed that three of the CopB residues (residues Y88, H89, and Y355) are in nearly the same configuration as in FrpB, thus they likely have an iron-binding function (Figures 6A,B).

Two of the residues (H89 and Y355) equivalent to the residues shown to be involved in iron binding in the published study (Saleem et al., 2013) were targeted for mutagenesis. Three strains with different site-directed mutations to these residues were made in order to determine if iron binding is important for CopB function. All three mutants (H89A, H89A/Y335F, and Y355F) grew on hTf and hLf on BHI containing 50μg/ml at a reduced amount compared to the wild type (Figure 6C) indicating they had reduced capacity to facilitate uptake of iron by TbpA or LbpA. When grown on plates containing 10μg/ml Desferal, these strains similarly did not form a lawn of growth on the entire plate similar to what was observed with the Δ*copB* strain. The expression of the mutant CopB proteins by the bacteria was confirmed by SDS-PAGE (Supplementary Figure S1).

### Are TonB-CopB Interactions Necessary for CopB-Facilitated Tf/Lf-Iron Utilization?

If TonB interactions are necessary for association of TBDTs near gaps in the peptidoglycan (Figure 5), then interruption of the TonB interaction would reduce the ability of CopB to facilitate Tf/Lf iron acquisition. Site-directed mutations of the TonB box in CopB were made to interfere with the interactions between CopB and TonB. The CopB TonB box was identified based on sequence comparison with the identified TonB box in other TBDTs. The pentapeptide DTVVS between residues 12 and 16 was predicted to be the TonB box of CopB and based on this prediction, three different TonB box mutants were made (Table 5). These modifications include the removal of the pentapeptide and replacing it with a single glycine residue (Δ12-16 mutant), the V14P mutant, and the V15P mutant.

**Table 5:** Growth phenotype of the strains with mutations in the CopB TonB box region.

Strain	WT	Δ*copB*	CopB Δ12–16	CopB V14P	CopB V15P
TonB box sequence	DTVVS	Absent	G----	DTPVS	DTVPS
Growth on hTf	++	−	−	−	++
Growth on hLf	++	+	+	+	++

++, denotes wild type growth level; +, denotes less than wild type growth level; −, denotes no growth. The strains were either plated onto BHI agar with 50μg/mL of Desferal. Around 3.1nmol of hTf, hLf, pTf, and buffer control was spotted onto the plate.

The detergent-solubilized protein extracts of each of constructed mutants were analyzed by SDS-PAGE to verify that the mutations did not significantly alter *copB* expression (Supplementary Figure S2). The presence of a ~75kDa band can be seen in Supplementary Figure S2 in the strains that had either a wild type or a mutant copy of the *copB* gene, while the band is absent from the CopB knockout strain. We performed the modified disk feeding assay on the three TonB box mutant strains (Table 5). Both the Δ12-16 mutant and V14P mutant had reduced growth on hTf/hLf similar to the CopB mutant. However, the V15P mutant did not affect ability of the bacteria to use hTf/hLf as iron sources (Table 5). All strains did not grow on the pTf control (data not shown). These results clearly demonstrate that interaction of CopB with TonB is required to facilitate the acquisition of iron from hTf and hLf.

### The Role of the REEF Domain in CopB-Facilitated Tf/Lf Iron Utilization

**Figure 7:**
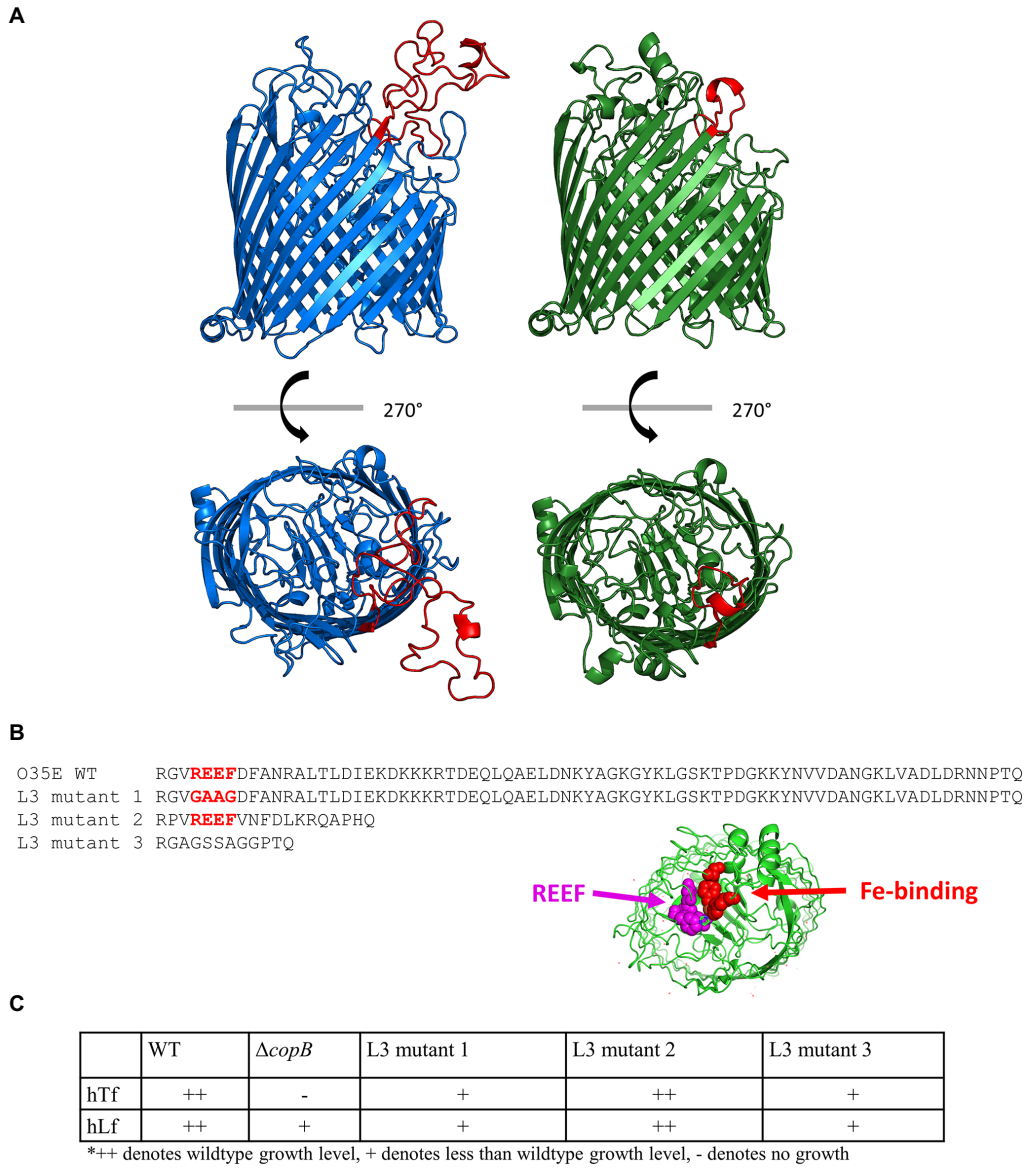
Role of REEF domain in loop 3. **(A)** Homology models of CopB from M. catarrhalis strain O35E (blue) and strain 417:082 (green). Extracellular loop 3 is shown in red. **(B)** Sequences of extracellular loop 3 from O35E WT, L3 mutant 1, L3 mutant 2, and L3 mutant 3. Location of REEF and Fe-binding residues. **(C)** hTf, hLf, and pTf growth phenotypes of CopB loop 3 mutants.

CopB is present in most isolates of *M. catarrhalis* and has a high sequence identity amongst various *M. catarrhalis* strains in the plug domain and the β-barrel. There is more sequence variation in the extracellular loops. There also is a dramatic difference in size of loop 3 among strains of *M. catarrhalis* with most strains having a large loop 3 and a few strains having a relatively small loop (Figure 7A; Liu et al., 2006). Extracellular Loop 3 is 74 residues long in strain O35E while it is only 18 residues long in strain 417:082. There is a conserved REEF motif in a portion of this loop that is shared with homologues in *N. meningitidis* and *M. bovis*. The portion of loop 3 where these residues are located is within the upper cavity of the beta-barrel with the side chains being adjacent to the residues involved in binding iron in the holo FrpB structure and postulated to be involved in iron uptake (Figure 6B; PDB ID: 4AIQ; Saleem et al., 2013).

In order to evaluate the potential role of this loop in CopB function, we designed and produced three different mutant CopBs and introduced them into strain O35E and tested their ability to facilitate iron acquisition from Tf/Lf. The first mutant CopB was generated by modification of the REEF motif to GAAG in the large loop 3 (Figure 7B) that resulted in the production of a stable protein (Supplementary Figure S3, lane d) but a substantial reduction in iron acquisition from hTf and hLf (Figure 7C). The second mutant simply involved replacing loop 3 in strain O35E with the smaller loop from 417:082 (Figure 7B). This resulted in the production of a smaller but stable protein (Supplementary Figure S2, lane c) but did not alter the ability of strain O35E to acquire iron from hTf or hLf (Figure 7C). A third mutant was constructed by linking the anchoring residues of loop 3 with a synthetic connecting peptide (Figure 7B) that generated a stable protein (Supplementary Figure S3, lane e) but also resulted in a substantial reduction in iron acquisition (Figure 7C).

## Discussion

One of the challenges in characterizing the iron acquisition pathways in bacteria like *M. catarrhalis* that primarily reside on the mucosal surfaces of the upper respiratory tract is that we have a very incomplete understanding of the physiological iron sources. Clearly there will be some environmental iron sources that are in particles trapped on the mucosal surface during inhalation. In this aerobic environment, it is likely that there will be ferric hydroxide clusters of varying sizes and little free ferric ion on many of these particles. The presence of specific receptors for Tf, Lf and heme iron sources in bacteria that colonize the upper respiratory tract indicate that there are also host sources of iron that might be available sporadically (nose bleeds) or could even be intentionally secreted on to the mucosal surface (Cavet et al., 2015; Lemire et al., 2015). Siderophores and other organic compounds produced by transient microbes or residents of the microbial community will be able to convert some of the environmental and host sources of iron to complexes with siderophores or other organic compounds to be used by microbial community. The relatively high level of expression of CopB and its homologues, FetA/FrpB and IrpA, under iron-limited conditions suggest that they may be important for acquiring iron from sources available on the mucosal surface. The ability of pathogenic *Neisseria* species to use ferric enterobactin by virtue of FetA/FrpB and the periplasm to cytoplasm pathway in its operon (Carson et al., 1999) suggests that it may be a potential source of iron on the mucosal surface. Although this iron-siderophore complex could readily be neutralized by the production of host siderocalin (Nelson et al., 2005), this may not apply to the extended spectrum of catecholate xenosiderophores that can be transported by this system (Hollander et al., 2011). The observation that the binding affinity for iron complexed to enterochelin by FetA was several orders of magnitude lower than that of the *Escherichia coli* FepA receptor (Carson et al., 1999) could indicate a different binding mechanism. Typical siderophore receptors have high affinity, specific binding that primarily recognizes the siderophore component whereas the periplasmic binding proteins recognize the iron coordinating residues and are capable of recognizing siderophores with considerable chemical and structural diversity (Clarke et al., 2002). The conserved residues in FetA and its homologues (Figure 5; Saleem et al., 2013) indicate that their binding mechanism is focused on iron, is similar to the periplasmic binding proteins, and thus is likely capable of utilizing a more diverse range of iron sources.

The ability of CopB and its homologues to potentially use a broader range of iron sources perhaps explains why the growth on BHI plates supplemented with 10μg/ml Desferal was dramatically reduced by deletion of the *copB* gene (Table 3) or by mutation of the critical iron-binding residues. The iron sources may include small ferric hydroxide clusters and ferric ion associated with a diverse array of organic molecules which to some extent may reflect the situation present on the mucosal surface. Clearly the availability of these diverse iron sources on mucosal surfaces would likely be considerably less than in BHI media. These iron sources would not likely be available at Desferal levels above 20μg/ml. We attempted to use ferric enterobactin as an iron source for *M. catarrhalis* on plates containing 50μg/ml Desferal, but were not able to observe growth on preparations with up to 50% iron saturated (data not shown). The experimental conditions were substantially different from those used to demonstrate use of ferric enterobactin by FetA (FrpB; Hollander et al., 2011); so we cannot really comment on any functional differences between CopB and FetA (FrpB).

More recently, it has been demonstrated that addition of aerobactin, enterobactin and salmochelins, but not other siderophores, to defined medium containing bovine Tf supported the growth of *N. gonorrhoeae* (Strange et al., 2011). Surprisingly this growth enhancement was not dependent on pilQ, MtrC, MtrD, or TonB, but was dependent upon the FbpABC periplasm to cytoplasm transport pathway. The implication clearly is that there may be multiple routes for these siderophores or their breakdown products to cross the outer membrane in a TonB-independent process. It also demonstrates that the laboratory measured binding constants for siderophores and the binding proteins do not necessarily predict iron transfer under physiological conditions. Although Desferal was not included in the extensive collection of xenosiderophores tested (Strange et al., 2011), our results suggest that it is not mediating TonB-independent iron acquisition from our Tf and Lf sources of iron since the TonB mutants were equivalent to a CopB deletion (Table 5).

The original demonstration that insertional inactivation of the *copB* gene reduced the ability of *M. catarrhalis* to utilize hTf and hLf (Aebi et al., 1996) could not readily be explained by the current model of TBDT from TbpA and LbpA (Noinaj et al., 2012, 2013). It was deemed important to first verify that this is not an artifact from the remaining ~1.3kb fragment of the *copB* gene producing the CopB plug domain and competing with other TBDTs for access to TonB (Figure 1). The reduced ability to utilize hTf and hLf was also observed in a strain deleted in the entire *copB* gene (Figure 4), confirming that CopB was indeed facilitating growth on exogenous hTf and hLf in iron depleted media. There appeared to be a trend for the insertional mutant to result in a greater reduction in growth than the complete knockout, suggesting that the plug domain may also be affecting the iron acquisition process, but the results were not statistically significant.

An obvious question regarding the phenomenon by which CopB impacts the utilization of iron from hTf and hLf is whether it can remove iron from hTf or hLf directly or whether it somehow enhances the efficiency of iron removal by these receptors (Figure 5). The direct utilization of iron from hTf and hLf by CopB/FetA might infer direct binding, but this was not observed in the solid phase binding assays with labeled hTf or hLf on whole cells from pathogenic *Neisseria* (Irwin et al., 1993; Biswas and Sparling, 1995) or *M. catarrhalis* (Bonnah et al., 1999) lacking TbpB/TbpA or LbpB/LbpA. As this might be a consequence of non-physiological binding conditions, the co-isolation of CopB with LbpA from *M. catarrhalis* with an hLf-Sepharose affinity resin under low stringency (more physiological) conditions (Bonnah et al., 1998) suggests that it binds hLf under physiological conditions. However, it is likely that this binding is mediated by the cationic N-terminal region of hLf that would not necessarily reflect a functional association and certainly would not likely occur with TbpA as well. Even if direct binding of hLf or hTf did occur, it is unlikely to be able to induce the conformational changes in hTf or hLf required to facilitate the iron removal mediated by TbpA or LbpA (Yadav et al., 2019).

Perhaps the most compelling evidence that CopB enhances the efficiency of the iron removal process (Figure 5) is that its impact on iron utilization from hLf is maintained at increasing levels of Desferal but clearly cannot mediate direct iron utilization at 20μg/ml Desferal or above (Table 3). There may be gradual release of iron from Tf and Lf by Desferal after they are applied onto the plate so that the speed and efficiency of the TonB-mediated iron removal directly from these iron sources may be critical. TonB-dependent processes would be very inefficient if it required chance association at gaps in the peptidoglycan layer of a TonB complex that was laterally diffusing through the inner membrane with TBDT receptors laterally diffusing in the outer membrane. Clearly there likely is a tendency for the TonB complex to be associated with gaps in the peptidoglycan layer where TBDTs may also tend to associate in order for efficient TonB-mediated transport to occur (Figure 5).

Previous studies have demonstrated that the lipoprotein RmpM forms complexes with LbpA, TbpA and FrpB, that these TBDTs tend to form oligomers in the outer membranes and that mutation of RmpM reduces iron-dependent growth with hLf (Prinz and Tommassen, 2000). This provides precedence for an indirect effect on TonB-mediated iron acquisition and support for the model illustrated in Figure 5. However, there are many unanswered questions that will require substantial effort to address. Does the synthesis of a TonB complex induce degradation of the peptidoglycan or are there pre-existing gaps that the TonB complexes migrate to and remain associated due to the TonB-mediated interactions with the TBDTs? The published work (Prinz and Tommassen, 2000) supports the concept of association of TBDTs at the gaps in the peptidoglycan layer due to the presence of lipoprotein homologues of RmpM. Structural studies with FrpB provided a symmetric trimer (PDB ID: 4AIQ; Saleem et al., 2013) suggesting there may be a natural tendency to self-associate. This could also promote the association of TBDTs at gaps in the peptidoglycan and indirectly affect TBDT.

The unique impact that CopB has on acquisition of iron from hTf and hLf prompted us to look for conserved residues and motifs that can be mutated to determine whether they are required for the enhanced growth on hTf and hLf. The structural comparison between the CopB homology model and the iron-holo *N. meningitidis* FrpB (FetA) structure identified putative CopB iron-binding residues (Figure 6B). The results showed that mutation of these residues affects iron utilization from hTf/hLf (Figure 6C) and from the BHI medium with 10μg/ml Desferal (Figure 6D). These results suggest that the lack of iron-binding residues reduces the formation or maintenance of the TBDT-TonB complexes illustrated in Figure 5 and indirectly reduces the efficiency of iron removal by TbpA and LbpA. One can also consider whether changes to the binding residues affect the transport of other iron sources by CopB that contribute to reaching a threshold of iron required to attain visible growth.

The CopB extracellular loop 3 has a high sequence diversity amongst various strains (Liu et al., 2006) and this diversity is most apparent between strains O35E and 417:082 (Figure 7B) as the latter strain has a dramatically reduced size of the loop. The transplantation of the strain 417:082 loop 3 into the strain O35E CopB had no effect on the growth on hTf/hLf, suggesting the residues that constitute the extended portion of loop 3 do not participate in iron utilization. Mutating the REEF residues of loop 3 or replacing it with a synthetic loop lacking these residues (Figure 7B) was sufficient for altering hTf/hLf-iron utilization (Figure 7C). Interestingly, these residues are conserved in *N. meningitidis* FetA and the arginine residue of this tetrapeptide has been suggested in a prior study to contribute to the basic environment around the iron-binding site, facilitating iron binding (Saleem et al., 2013). It is likely that these residues are essential for binding or transport of iron through CopB and interaction with TonB.

For substrate uptake to occur, TBDTs must interact with TonB for energy transduction from the inner membrane proton motive force. This interaction has been well characterized in *E. coli* receptors and is mediated by the formation of a paired beta strand between the TonB C-terminal domain and the N-terminal TonB box of the receptor (Pawelek et al., 2006; Shultis et al., 2006). In this study, the deletion of the entire CopB TonB box and the mutation of the third residue of the TonB box from valine to proline affect hTf/hLf-iron utilization (Table 5), indicating that the effect of CopB is TonB-dependent. To further confirm this, the fourth residue of the TonB box was mutated from valine to proline, which showed no effect on iron utilization. This observation complies with prior *E. coli* TonB box studies, which showed the mutation of the fourth residue of the TonB box has a little to no effect on receptor function (Gudmundsdottir et al., 1989). Thus, the interaction between CopB and TonB is likely to have made iron acquisition from Tf and Lf through TbpA and LbpA more efficient (Figure 5).

The impact of CopB on iron acquisition from Tf and Lf, has also been demonstrated for its homologue, IrpA, in *M. bovis* (Kakuda et al., 2003) and although this has not been directly reported for the CopB homologue, FrpB/FetA, in *N. meningitidis*, the formation of the heterooligomeric complex with TbpA, LbpA, and RmpM and impact of the RmpM mutation suggests that this may occur in the *Neisseria* species (Prinz and Tommassen, 2000). Notably these proteins are expressed at higher levels than the other TBDTs in these species, indicating that they likely play an important role in acquisition of the forms of iron available under physiological conditions in the upper respiratory tract (or genitourinary tract) where they normally reside. The degree to which the effect of level of expression or the tendency to form trimeric complexes (Saleem et al., 2013) has an impact on acquiring iron from Tf and Lf is uncertain, but our experiments with the *tbpA* or *lbpA* gene replacing *copB* (Table 4) suggest that the inherent properties of the CopB protein may be important. However, the requirement for associating with TbpB/LbpB and the substantial conformational changes upon binding Tf/Lf may interfere with maintenance of stable complexes by TbpA or LbpA. These complexes may have an additional complexity due to the insolubility of the ferric ion that may require ready access by FbpA for efficient transfer of the ferric ion (Siburt et al., 2009).

## Data Availability Statement

The raw data supporting the conclusions of this article will be made available by the authors, without undue reservation.

## Author Contributions

AS and CC conceived and designed the study and wrote the manuscript. CC performed the experiments. DN analyzed the data and edited the manuscript. All authors contributed to the article and approved the submitted version.

## Funding

This work was supported by the funding from the Canadian Institutes of Health Research (CIHR) (grant number PJT 148804). CC was supported by graduate scholarships from CIHR and Alberta Children’s Hospital Research Institute.

## Conflict of Interest

The authors declare that the research was conducted in the absence of any commercial or financial relationships that could be construed as a potential conflict of interest.

## Publisher’s Note

All claims expressed in this article are solely those of the authors and do not necessarily represent those of their affiliated organizations, or those of the publisher, the editors and the reviewers. Any product that may be evaluated in this article, or claim that may be made by its manufacturer, is not guaranteed or endorsed by the publisher.
