# Supplementary material for: The Role of the Moraxella catarrhalis CopB Protein in Facilitating Iron Acquisition From Human Transferrin and Lactoferrin
Source: Front Microbiol. 2021 Sep 23;12:714815. doi: 10.3389/fmicb.2021.714815 (PMC8497027; doi:10.3389/fmicb.2021.714815)
Supplement: Supplementary file 1 [file Presentation_1.pdf]

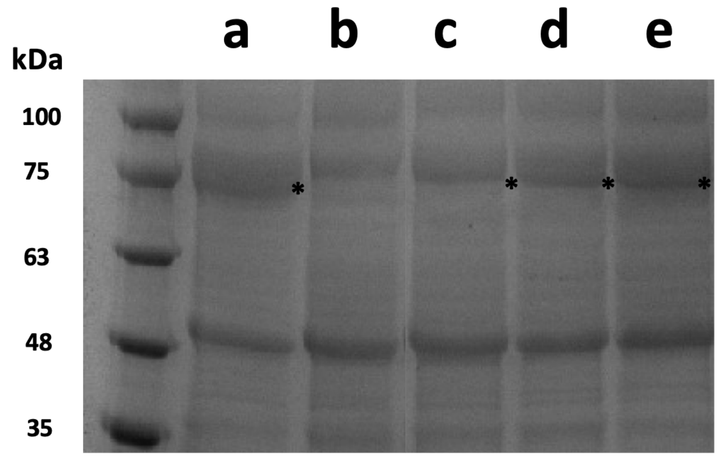

Figure S1. SDS-PAGE of detergent-solubilized membrane proteins from iron-starved cultures. Wildtype (a), Δ*copB* mutant (b), CopB H89A mutant (c), CopB H89A/Y355F mutant (d), and CopB Y355F mutant (e). The CopB protein is indicated by an asterisk.

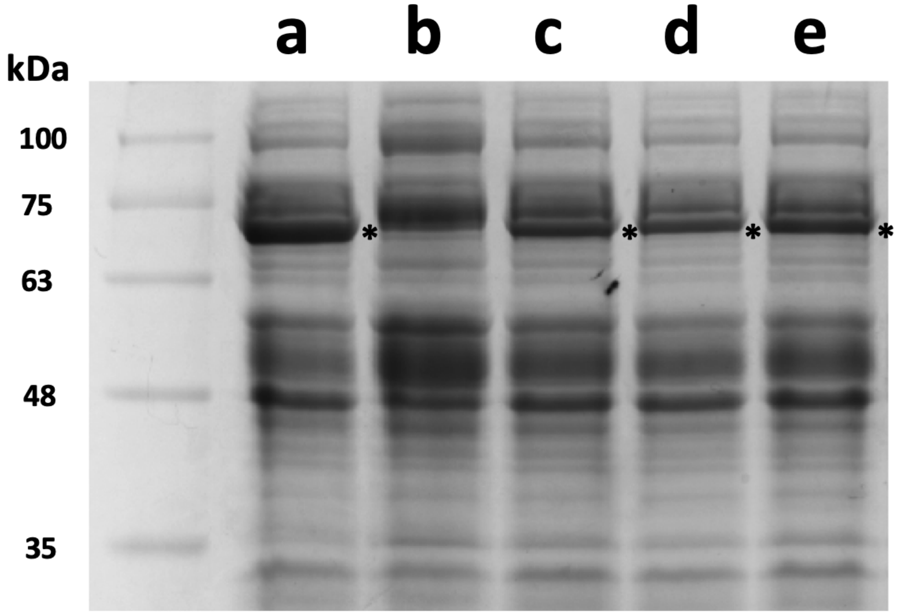

Figure S2. SDS-PAGE of detergent-solubilized membrane proteins from iron-starved cultures. Wildtype (a), Δ*copB* mutant (b), CopB Δ12-16 mutant (c), CopB V14P mutant (d), and CopB V15P mutant (e). The CopB protein is indicated by an asterisk.

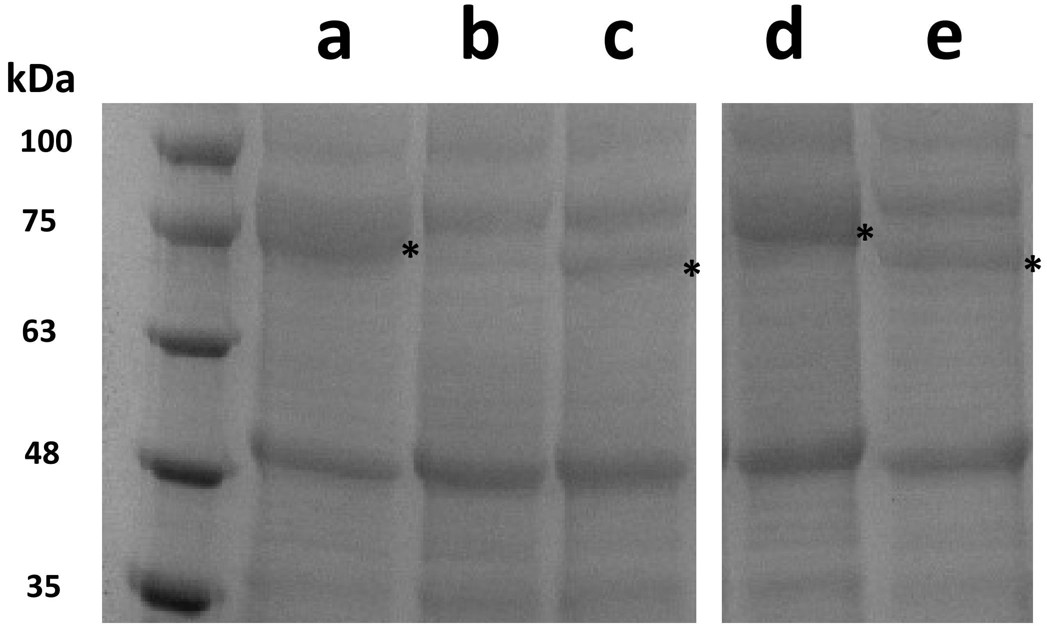

Figure S3. SDS-PAGE of detergent-solubilized membrane proteins from iron-starved cultures. Wildtype (a), Δ*copB* mutant (b), CopB L3 mutant 2 (c), CopB L3 mutant 1 (d), and CopB L3 mutant 3 (e). The CopB protein is indicated by an asterisk. This figure is a composite image of two different parts of the same gel.
